# IGF-1 enhances BMSC viability, migration, and anti-apoptosis in myocardial infarction via secreted frizzled-related protein 2 pathway

**DOI:** 10.1186/s13287-019-1544-y

**Published:** 2020-01-09

**Authors:** Mingzhuo Lin, Xinyue Liu, Haoxiao Zheng, Xiaohui Huang, Yu Wu, Anqing Huang, Hailan Zhu, Yunzhao Hu, Weiyi Mai, Yuli Huang

**Affiliations:** 1 Department of Cardiology, Shunde Hospital, Southern Medical University (the first people’s hospital of Shunde), Jiazhi Road, Lunjiao Town, Shunde District, Foshan, 528300 People’s Republic of China; 2 Department of Cardiology, The First Affiliated Hospital of Sun Yat-sen University, Guangzhou, 510080 People’s Republic of China; 3 The George Institute for Global Health, Sydney, Australia

**Keywords:** Bone marrow mesenchymal stem cell, Insulin-like growth factor-1, Myocardial infarction, Secreted frizzled-related protein 2

## Abstract

**Background:**

Bone marrow mesenchymal stem cell (BMSC) transplantation represents a promising therapeutic strategy for ischemic heart disease. However, its effects are hampered by the poor viability of transplanted cells and the hostile microenvironment of the ischemic region. Insulin-like growth factor-1 (IGF-1) is an important paracrine growth factor of BMSC and plays an important role in the properties of BMSC. Here, we investigated whether overexpressing IGF-1 could enhance the BMSC viability, migration, anti-apoptosis, and protective effects of cardiomyocytes, and explore the underlying mechanisms’ focus on the role of the AKT/secreted frizzled-related protein 2 (SFRP2)/β-catenin pathway.

**Methods:**

We constructed BMSCs overexpressing insulin-like growth factor-1 (BMSCs-IGF-1) or empty vector (BMSCs-NC) using lentivirus, and evaluated cell survival, proliferation, and migration under normoxic and hypoxic conditions. Co-culture of rat cardiomyoblasts with BMSCs was performed to explore the paracrine effect of BMSCs-IGF-1 for rescuing cardiomyoblasts under hypoxia. Transplantation of BMSCs in acute myocardial infarction rats was used to explore the effect of BMSCs-IGF-1 therapy.

**Results:**

BMSCs-IGF-1 exhibited a higher cell proliferation rate, migration capacity, and stemness, and were more resistant to apoptosis under hypoxia. Overexpression of IGF-1 upregulated the expression of total and nuclear β-catenin via the AKT-secreted frizzled-related protein 2 (SFRP2) pathway, which enhanced cell survival. Inhibition of AKT or *SFRP2* knockdown by siRNA significantly antagonized the effect of IGF-1 and decreased the expression of β-catenin. The expression of β-catenin target genes, including cyclin D1 and c-Myc, were accordingly decreased. Moreover, BMSCs-IGF-1 could rescue cardiomyoblasts from hypoxia-induced apoptosis and preserve cell viability under hypoxia. Transplantation of BMSCs-IGF-1 into myocardial infarction rats greatly reduced infarct volume than BMSCs-NC, with significantly greater expression of SFRP2 and β-catenin.

**Conclusions:**

These results suggest that in BMSCs overexpressing IGF-1, SFRP2 is an important mediator for the enhancement of stem cell viability via activating, rather than antagonizing, the Wnt/β-catenin pathway.

## Background

Acute myocardial infarction (AMI) and post-infarction chronic heart failure (CHF) are the leading causes of mortality worldwide [1]. Despite advancements in therapy for AMI and CHF during the past decades, it is necessary to find new therapeutics to decrease the morbidity and mortality from AMI. Stem cell transplantation is one of the attractive therapies for AMI [2]. Among various types of stem cells, bone mesenchymal stem cell (BMSC) transplantation represents a promising therapeutic strategy [3–5]. However, the effects of stem cell therapy are modest and hampered by the poor viability of the transplanted cells. It has been reported that most engrafted BMSCs lose viability within 3 days of transplantation due to the hostile microenvironment of the ischemic region [6]. As a result of insufficient blood flow, the ischemic tissues suffer from hypoxia, insufficiency of metabolic substrates, and accumulation of metabolic waste, which impairs the survival of engrafted cells. Therefore, it is important to reinforce BMSC’s resistance to hypoxia.

Genetically engineered BMSCs overexpressing AKT (BMSCs-AKT) are more resistant to hypoxia with a preserved normal metabolism, and survive in the myocardium [7]. Secreted frizzled-related protein 2 (SFRP2) is a major paracrine mediator of BMSCs-AKT myocardial survival and reparative effects [8]. Insulin-like growth factor-1 (IGF-1) is an important paracrine growth factor of BMSC [9]. Allahdadi et al. showed that overexpression of IGF-1 in BMSCs resulted in increased cell survival, immunomodulation, and functional improvements in spinal cord injury [10]. Our previous studies showed that IGF-1, an upstream activator of AKT, is an important paracrine growth factor of BMSCs [11], and it increases the migration and proliferative viability of BMSCs in a dose-dependent manner [12]. However, the underlying mechanisms have not been fully elucidated, and whether these effects are mediated by AKT/SFRP2 requires further exploration. Furthermore, the Wnt/β-catenin pathway has an important role in the development of stem cells and control of their properties [13, 14]. SFRP2 has generally been considered as an antagonist of the canonical Wnt/β-catenin pathway [15]. However, other studies have found that SFRP2 may also enhance Wnt-mediated signaling [16, 17]. Nonetheless, the interaction of SFRP2 with the Wnt/β-catenin pathway in BMSCs is unknown.

Based on the beneficial effects of IGF-1, we hypothesized that overexpression of IGF-1 in BMSCs could enhance stem cell viability, migration, and the effects of anti-apoptosis. It also strengthens the effects of anti-apoptosis in cardiomyocytes under hypoxic conditions in vitro. These synergic effects will result in better cardiac repair and regeneration after AMI in vivo. To explore the underlying mechanisms, we examined the role of the AKT/SFRP2/β-catenin pathway in IGF-1-mediated enhanced viability, migration, and anti-apoptosis in BMSCs.

## Methods

### Cell culture

BMSCs were flushed from the femurs and tibiae of SD rats with α-minimum essential medium (α-MEM; Thermo Fisher Scientific, Waltham, MA, USA) containing 20% fetal bovine serum (FBS; Gibco, Grand Island, NY, USA). Cells were collected and seeded in flasks with α-MEM supplemented with 10% FBS and incubated at 37 °C in a humidified atmosphere of 5% CO_2_. The medium was changed every 3 days. To identify the cell phenotype, flow cytometry analysis was performed to examine cell surface markers CD73, CD105, CD90, and CD45 (CD73, eBioscience, 11-0739-41; CD105, eBioscience, 12-1051-81; CD90, eBioscience, 11-0900-81; CD45, eBioscience, 11-0460-82, respectively) according to the recommendation of the International Society for Cellular Therapy [18]. After 2–3 passages, BMSCs negative for CD45 and positive for CD73, CD105, and CD90 were used.

H9C2 rat cardiomyoblasts were purchased from the Cell Bank of Chinese Scientific Academy (Shanghai, China) and maintained in Dulbecco’s modified Eagle’s medium (DMEM; Gibco, Grand Island, NY, USA) supplemented with 10% FBS.

### IGF-1-overexpressing BMSC (BMSCs-IGF-1) generation

The cDNA of rat IGF-1 (*IGF1*) was PCR-amplified (forward, TGCTCTAGAATGAGCGCACCTCCAATAAAGATA; reverse, CGGAATTCTCTACTTGTGTTCTTCAAGTGTACTTCC) and cloned into the *EcoRI* and *XbaI* sites of the LV-003 lentivirus vector (Forevergen Biosciences Center, Guangzhou, China). The lentivirus vector was co-transfected with packaging vectors into 293T cells to produce recombinant lentivirus. BMSCs were exposed to recombinant lentivirus and cultured in a medium with 2 μg/mL puromycin to generate BMSCs-IGF-1 and BMSCs expressing an empty vector (BMSCs-NC). To confirm the overexpression of IGF-1, BMSCs-NC and BMSCs-IGF-1 were seeded at a density of 1 × 10^6^ cells per 10 cm dish. Thirty-six hours later, the supernatants were collected and subjected to an enzyme-linked immunosorbent assay (ELISA) (CUSABIO ELISA Kit, CSB-E04582r) according to the manufacturer’s protocol.

### Hypoxia protocol and normoxia control

Hypoxic conditions were created using AnaeroPack-Anaero (Mitsubishi, Tokyo, Japan). The culture flasks containing cells were placed in a 7-L anaerobic jar (Mitsubishi, Tokyo, Japan) with three sachets of AnaeroPack-Anaero and were incubated at 37 °C for 48 h. The O_2_ concentration in the jar was expected to decrease to less than 1% within 1 h. Hypoxia was terminated by opening the anaerobic jar and removing the cells. The cells of the normoxia control were maintained in a 37 °C regular culture incubator with 5% CO_2_ during preparation.

### 3-(4,5-Dimethylthiazol-2-yl)-5-(3-carboxymethoxyphenyl)-2-(4-sulfophenyl)-2h-tetrazolium (MTS) assay

Cells were seeded in 96-well plates at a density of 1 × 10^4^ cells per well and were maintained for 24 h at 37 °C in 5% CO_2_. Then, an MTS reagent (Promega; Madison, WI, USA, G1112) was added to each well and incubated for 4 h. Absorbance was monitored using a plate reader (Diatek) at OD = 490 nm for 3 days to evaluate cell proliferation. All samples were assayed in triplicate.

### Terminal deoxynucleotidyl transferase-mediated dUTP nick-end labeling (TUNEL) assays

The cells were seeded onto slides, and the TUNEL assays were performed to detect apoptotic cells according to the protocol provided by the DeadEnd™ Fluorometric TUNEL System Kit (Promega, Madison, WI, USA). Apoptotic cells were labeled with fluorescein-12-dUTP, resulting in localized green fluorescence within the nuclei that can be observed under a fluorescence microscope. For each sample, five non-overlapping fields at × 100 magnification were randomly captured using a video camera, and the number of TUNEL-positive cells was counted in a blind fashion.

### Annexin-V-fluorescein isothiocyanate (FITC) apoptosis assay

Apoptosis was quantified using Annexin-V-FITC (BD Biosciences, San Jose, CA, USA) and propidium iodide (PI, BD Biosciences, San Jose, CA, USA), according to the manufacturer’s instructions. A total of 1 × 10^3^ cells were analyzed by flow cytometry, and the data were analyzed using the CellQuest software (BD Biosciences, San Jose, CA, USA). Cells that were positive for Annexin-V-FITC were considered to be undergoing apoptosis.

### Migration assay

Cell migration assays were performed using Transwell culture inserts with 8-μm pore polyester membranes (Corning, 3422). BMSCs were seeded at a density of 1 × 10^5^ cells/100 μL/well in the upper chamber in serum-free medium, while medium containing 10% FBS was placed in the lower chamber. Following an incubation period of 24 h, the cells remaining on the upper chamber were removed, and the cells that had migrated through the membrane were fixed in methanol and stained with crystal violet. For each stained membrane, five non-overlapping fields at × 100 magnification were randomly captured with a video camera, and the number of migrated cells was counted. All cell lines were assayed in triplicate.

### Western blot analysis

Cells and fresh myocardial tissues from the myocardial infarction (MI) area were washed three times with cold PBS and lysed with the RIPA buffer (Beyotime, P0013B) containing protease and phosphatase inhibitors (Boster, AR1183) according to the manufacturer’s instructions. A BCA protein assay kit (Beyotime, P0011) was used to determine the protein concentration. Equal amounts of protein were loaded on the wells of each line in a 10% SDS-PAGE gel for electrophoresis and then transferred to a PVDF membrane. The membranes were then blocked with 5% BSA in TBST buffer for 1 h at room temperature, and incubated with a primary antibody against caspase-3 (CST, 9662s), BAX (CST, 2774), BCL-2 (Abcam,196495), OCT4 (Boster,bs-0830R), p-AKT (CST, 13038S), AKT (CAT, 2920), ERK1/2 (CST, 9102), p-ERK1/2 (CST, 4370S), p-β-catenin (ser33/37/T41, CST, 9561s), β-catenin (Santa, sc7199), SFRP2 (Abcam, ab86329), and NFAT4 (Abcam, ab93628) at 4 °C overnight. Following three washes with TBST, the membranes were incubated with their corresponding secondary antibody (Jackson 111-035-003 and Jackson 111-005-003) at room temperature for 1 h, and the blots were visualized using chemiluminescence (ECL; Forevergen Biosciences Center, Guangzhou, China). Blots were quantified by densitometric scanning. The level of protein expression was normalized against GAPDH controls.

### Co-culture of H9C2 rat cardiomyoblast cells and BMSCs

For co-culture experiments, BMSCs-NC or BMSCs-IGF-1 were seeded onto the lower chamber of the apparatuses (Corning, 3412) at 5 × 10^5^ cells/well, 60 μL/well. The cells were incubated overnight at 37 °C in 5% CO_2_. Subsequently, 3 × 10^5^ H9C2 cells were seeded onto the upper chamber. After a normal culture of 24 h, the co-cultured cells were exposed to hypoxia as described above. H9C2 cells were collected for apoptosis assay and Western blot analysis. For the MTS assay, BMSCs-NC or BMSCs-IGF-1 were harvested in the lower chambers. H9C2 cell viability was determined at 1, 2, and 3 days post-reoxygenation as described above.

### Immunofluorescence (IF) staining

For IF staining, BMSCs-NC or BMSCs-IGF-1 were seeded onto culture slides and subjected to a hypoxic treatment. The slides were fixed in 4% paraformaldehyde (PFA) and then treated with 0.1% Triton X-100. The slides were blocked by 5% BSA at room temperature for 1 h and then incubated with the primary antibody against β-catenin (1:50, Santa Cruz) overnight at 4 °C. Following three washes with PBS, the slides were incubated with Alexa Fluor 488 donkey anti-mouse IgG (1:500; Invitrogen) for 1 h at room temperature. After washing three times with PBS, the slides were stained with Hoechst (1:1000; Invitrogen) and mounted, and then photographed under a fluorescence microscope.

### Transfection and LY294002 treatment

si-*SFRP2* (CCGAAAGGGACCTGAAGAATT) was purchased from GenePharma (Shanghai, China). Cells were seeded into 6-well plates 12 h prior to transfection. A final concentration of 100 nM siRNA was transfected into the cells using Lipofectamine 2000 (Invitrogen, Carlsbad, CA, USA) according to the manufacturer’s protocol. At 48 h post-transfection, the cells were exposed to hypoxia or normoxia as described above. The cells were then subjected to MTS, migration, and Western blot assays.

LY294002 was purchased from Selleck (S1105). Cells were treated with either 0.5 μM LY294002 or an equal volume of DMSO as vehicle control for 24 h, and then were exposed to hypoxia or normoxia as described above. The cells then underwent MTS, migration, and Western blot assays.

### Myocardial infarction (MI) model and cell transplantation

All animal experiments and surgical procedures were approved by the Shunde Hospital of Southern Medical University (approval number: SDRMYY#0002). According to the standards of the National Institutes of Health, all animals received humane care, and all procedures were strictly carried out under best practices. Ligation of the left anterior descending coronary artery was performed on adult SD rats (approximately 200 g) for the animal model of MI. Briefly, the rats were anesthetized and ventilated with room air using a small animal ventilator, and then, the left anterior descending coronary artery was ligated with a 6–0 silk suture midway between the left atrium and the apex of the heart. Successful performance of coronary artery occlusion was verified by a change in color of the myocardium distal to the coronary ligation. Then, the wound was sutured immediately. Sham animals underwent placement of the suture without ligation.

For cell transplantation, about 1 × 10^6^ BMSCs-NC or BMSCs-IGF-1 were suspended in 1.5 mL serum-free medium. One week after coronary ligation, suspensions containing BMSCs were injected via the tail vein. MI rats were randomly divided into three groups (six rat each) that received BMSCs-NC, BMSCs-IGF-1, or serum-free medium control. The sham rats were injected with a serum-free medium via the tail vein. Three weeks after BMSCs transplantation, the left ventricular internal diameter at end diastole (LVIDd), left ventricular anterior wall during diastole (LVAWd), ejection fraction (EF), fractional shortening (FS), stroke volume (SV), and cardiac output (CO) were assessed using an ACUSON Sequoia 512 Ultrasound System (Siemens Medical, Erlangen, Germany). Animals were sacrificed with an overdose of potassium chloride. Tissues with ischemic areas were harvested for Western blotting and histological analysis.

### Histological analysis

Tissues were fixed in formalin and embedded in paraffin, and then cut into 4-μm sections. To evaluate the survival of BMSCs in the infarcted myocardium, the sections were stained with DAPI (1:1000; Invitrogen), and then photographed under a fluorescence microscope for detection of GFP-positive cells. To analyze the infarction size, sections were subjected to Masson’s trichrome stain according to the manufacturer’s instructions (Trichrome stains Masson Kit, Sigma-Aldrich, St. Louis, MO, USA). Size was expressed as a percentage of the total ventricular area.

For immunohistochemistry (IHC), the sections were deparaffinized and rehydrated, and then subjected to heat-induced epitope retrieval. Following blocking with 3% hydrogen peroxide, the sections were incubated with primary antibodies against BAX (1:100, CST, 2774), BCL-2 (1:100, Abcam, 196495), and caspase-3 (1:50, CST, 9662s) at 4 °C overnight. Subsequently, the sections were washed three times with PBS, followed by incubation with either polymerization HRP-conjugated anti-rabbit IgG (Boster, Wuhan, China) or polymerization HRP-conjugated anti-mouse IgG (Boster, Wuhan, China) for 1 h at room temperature. The sections were visualized using a DAB Chromogenic Substrate Kit (Boster, Wuhan, China) and hematoxylin counterstain solution (Boster, Wuhan, China) according to the manufacturer’s protocol.

### Statistical analysis

All experiments were performed at least three times. GraphPad Prism v.6 (GraphPad Software Inc., La Jolla, CA, USA) was employed for the statistical analysis. The results are presented as mean ± SD. Data across three and more groups were assessed by the chi-square test, and if significant differences were observed, we further performed pairwise comparison between two groups using Student’s *t* test. The differences were considered significant at a *P* value of < 0.05.

## Results

### Overexpression of IGF-1 protects BMSCs against hypoxia

**Fig. 1:**
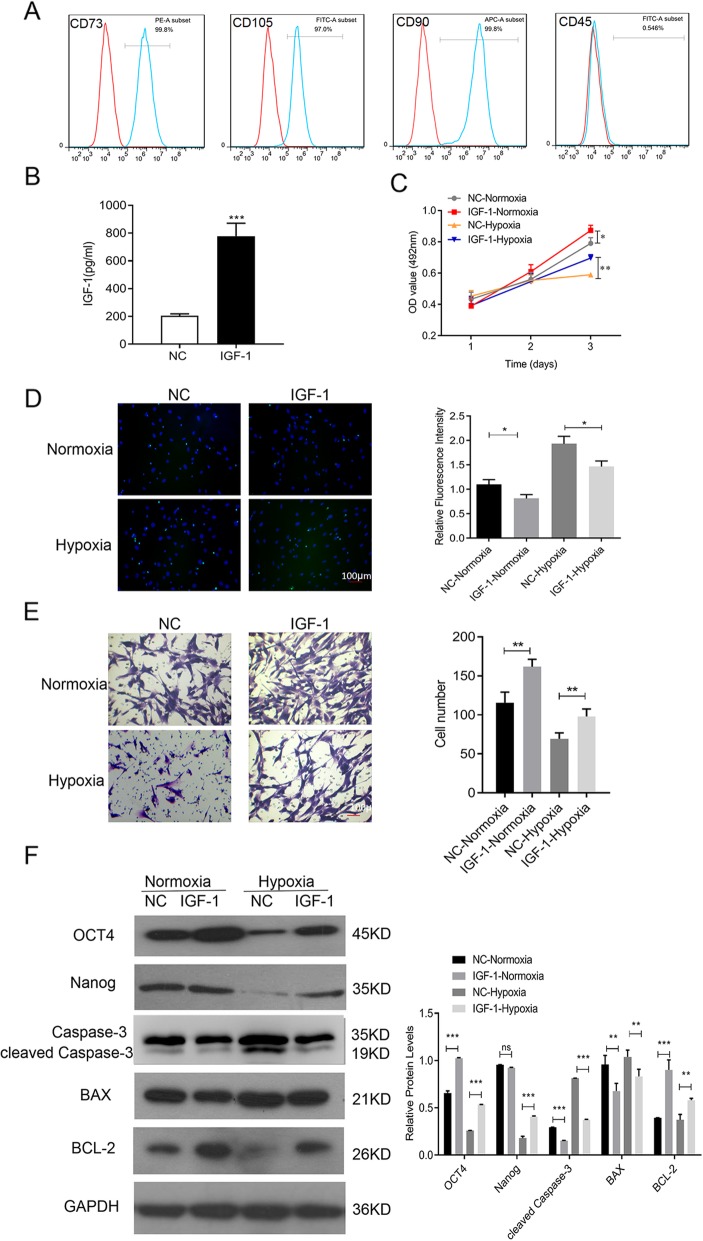
Effect of IGF-1 overexpression on BMSCs. **a** Identification of BMSCs by flow cytometry analysis. **b** Supernatants from cultured BMSCs-NC and BMSCs-IGF-1 were collected and subjected to ELISA to determine IGF-1 levels. **c** Cells were exposed to hypoxia for 48 h, and cell proliferation was determined by MTS assay. **d** Apoptosis was determined by TUNEL assay. **e** Cell migration was determined by Transwell assay. **f** Expression of OCT4, NANOG, cleaved caspase-3, BAX, and BCL-2 was determined by Western blotting. All assays were performed in triplicate (*P < 0.05, **P < 0.01, ***P < 0.001)

Typical BMSCs were either small and spindle- or triangular-shaped, and characteristically adhered to the surface of culture dishes. Flow cytometry analysis showed that over 90% of cells were positive for CD73, CD105, and CD90 surface markers, but not CD45 (Fig. 1a). CD73, CD105, and CD90 are considered as surface markers of undifferentiated BMSCs. The lack of CD45 cell surface antigens indicated that BMSCs were successfully distinguished from hematopoietic stem cells. Therefore, the cells employed in this study were regarded as BMSCs.

To construct genetically engineered BMSCs overexpressing IGF-1 (BMSCs-IGF-1), lentivirus was used to transduce BMSCs with IGF-1. The level of IGF-1 in the medium secreted from BMSCs-IGF-1 was determined by ELISA, which was fourfold (95% confidence interval 2.52–6.35) higher than BMSCs-NC (empty vector) (Fig. 1b).

To explore the role of IGF-1 overexpression in BMSCs under hypoxia, BMSCs-IGF-1 and BMSCs-NC were exposed to hypoxia for 48 h, and cell viability was monitored for 3 days by an MTS assay. As shown in Fig. 1c, IGF-1 promoted BMSCs proliferation in normoxia. Under hypoxia, BMSCs proliferation was inhibited, but this was significantly attenuated by the overexpression of IGF-1. Hypoxia-induced apoptosis in BMSCs-IGF-1 and BMSCs-NC was evaluated by TUNEL staining. Our results indicated that IGF-1 reduced hypoxia-induced apoptosis by 25% (95% confidence interval 11–37%, Fig. 1d). A separated and merged image of TUNEL assay is also presented in Additional file 1: Figure S1. Transwell assays were performed to evaluate the effects of IGF-1 overexpression on cell migration. Images of migration are shown in Fig. 1e. After exposure to hypoxia, the number of migrated BMSCs-IGF-1 was significantly more than BMSCs-NC. These findings indicated that BMSCs-IGF-1 exhibited greater migration ability under hypoxia compared with BMSCs-NC.

Moreover, the expression of pro-apoptotic cleaved caspase-3 and BAX, and anti-apoptotic BCL-2 was determined by Western blotting. Under normoxia, IGF-1 downregulated BAX, cleaved caspase-3, and upregulated BCL-2. As expected, in BMSCs-NC cells, cleaved caspase-3 and BAX were increased and BCL-2 decreased in response to hypoxia, whereas hypoxia only slightly decreased BAX, cleaved caspase-3, and increased BCL-2 in BMSCs-IGF-1 (Fig. 1f). These results suggested that BMSCs-IGF-1 had much higher resistance to hypoxia-induced apoptosis.

The expression of pluripotency-related transcription factors OCT4 and NANOG was detected by Western blotting [19, 20]. In normoxia, IGF-1 increased OCT4 expression. Under hypoxic conditions, OCT4 and NANOG were decreased in BMSCs, while IGF-1 overexpression could restore OCT4 and NANOG expression (Fig. 1f). These data implied that overexpression of IGF-1 maintained the stemness of BMSCs under hypoxia.

### BMSCs-IGF-1 protect H9C2 rat cardiomyoblasts against hypoxia

**Fig. 2:**
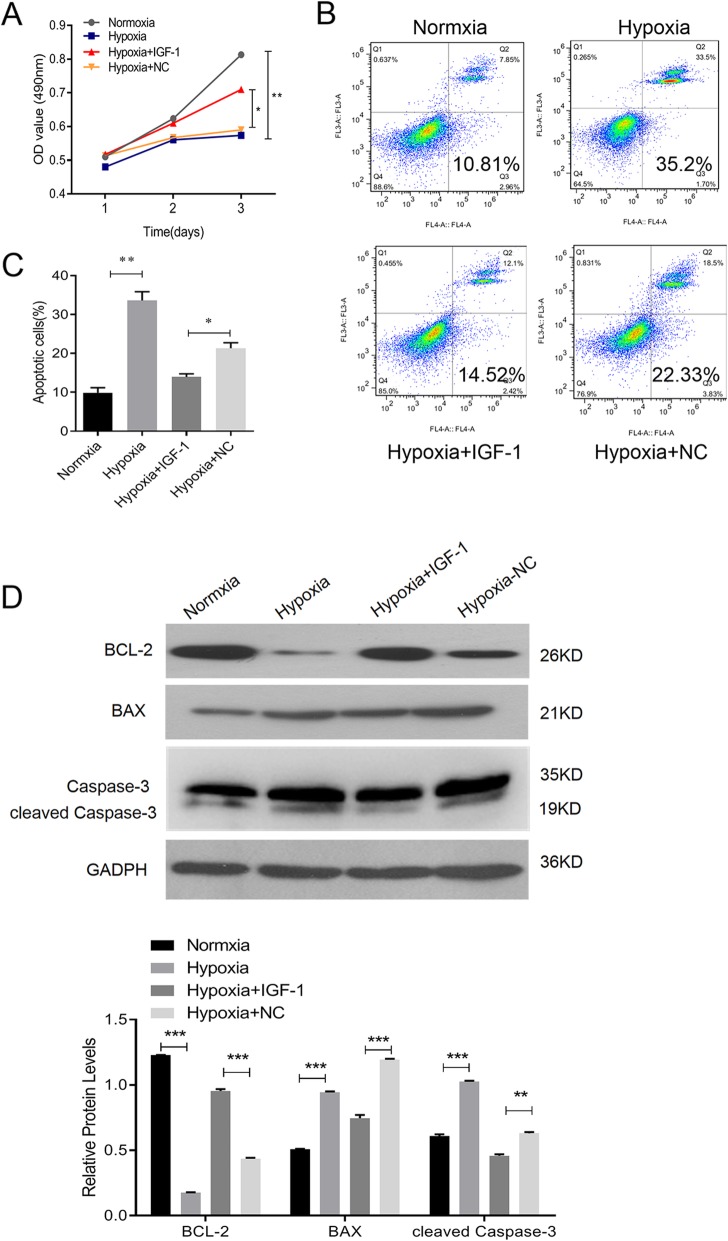
BMSCs-IGF-1 protected H9C2 rat cardiomyoblast cells against hypoxia. H9C2 were co-cultured with BMSCs-NC, BMSCs-IGF-1, or control medium, and then exposed to hypoxia for 48 h. **a** Cell proliferation of H9C2 was determined by MTS assay. **b**, **c** Apoptosis of H9C2 was determined by Annexin-V-FITC staining. **d** Expression of BCL-2, BAX, and cleaved caspase-3 in H9C2 was determined by Western blotting. All assays were performed in triplicate (*P < 0.05, **P < 0.01, ***P < 0.001)

To explore the effects of BMSCs-IGF-1 on cardiomyoblasts under hypoxia, H9C2 rat cardiomyoblasts were co-cultured with BMSCs-IGF-1, BMSCs-NC, or medium control, and the cells were exposed to hypoxia for 48 h. The proliferation and apoptosis of H9C2 were evaluated. The MTS results suggested that hypoxia inhibited the proliferation of H9C2, while co-culture with BMSCs-IGF-1 significantly rescued H9C2 from growth arrest (Fig. 2a). However, BMSCs-NC failed to preserve the viability of H9C2 cells under hypoxia (Fig. 2a). Apoptosis of H9C2 was detected with Annexin-V-FITC staining. As shown in Fig. 2b, there were fewer apoptotic cells (14.52 ± 1.58%) induced by hypoxia in H9C2 co-cultured with BMSCs-IGF-1 than with the medium control (35.2 ± 5.02%). Co-culture with BMSCs-NC had no effect on anti-apoptosis induced by hypoxia in H9C2 (22.33 ± 2.29%). Additionally, the pro-apoptotic proteins cleaved caspase-3, BAX, and anti-apoptotic protein BCL-2 were detected by Western blotting (Fig. 2c). Hypoxia decreased BCL-2 while increasing BAX and cleaved caspase-3 in H9C2. When BMSCs-IGF-1 were co-cultured with H9C2, the levels of BCL-2, BAX, and cleaved caspase-3 in hypoxic H9C2 were similar to the cells cultured under normoxia. In contrast, BMSCs-NC seeded onto the lower chamber only slightly downregulated BCL-2, upregulated BAX, and cleaved caspase-3 in H9C2 under hypoxia. These findings suggested that BMSCs-IGF-1 exerted more powerful protective effects on H9C2 survival under hypoxia compared with BMSCs-NC.

### BMSCs-IGF-1 exhibit higher levels of p-AKT, SFRP2, and β-catenin than BMSCs-NC

**Fig. 3:**
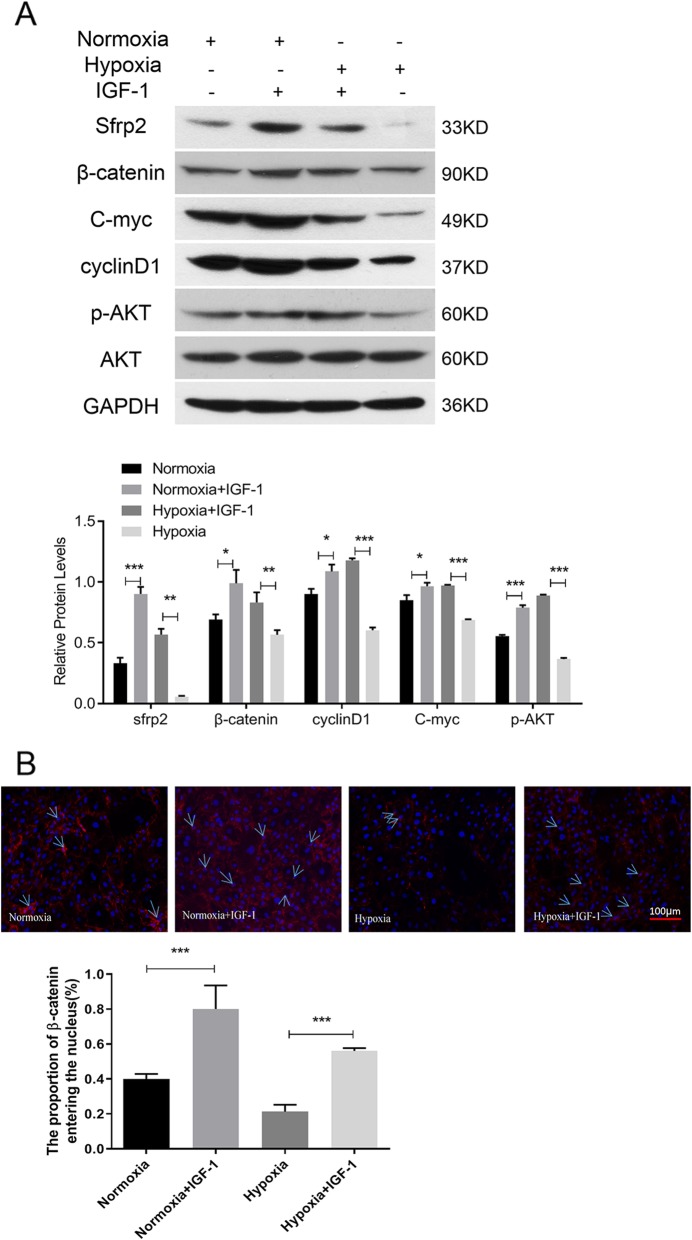
BMSCs-IGF-1 exhibited higher levels of p-AKT, SFRP2, and β-catenin than BMSCs-NC. **a** BMSCs-NC or BMSCs-IGF-1 were exposed to hypoxia for 48 h; expression of SFRP2, β-catenin, c-myc, cyclin D1, p-AKT, and AKT were determined by Western blotting. **b** Expression and localization of β-catenin were determined by immunofluorescence staining. All assays were performed in triplicate (*P < 0.05, **P < 0.01, ***P < 0.001)

AKT-modified BMSCs have been reported to mediate tissue repair through paracrine SFRP2 [21, 22]. Since IGF-1 is an upstream activator of the AKT pathway, AKT, p-AKT, and SFRP2 protein levels in BMSCs-IGF-1 and BMSCs-NC were examined under hypoxia and normoxia. As shown in Fig. 3a under both normoxia and hypoxia, IGF-1 dramatically stimulated the phosphorylation of AKT and the expression of SFRP2 in BMSCs. With hypoxia exposure, p-AKT and SFRP2 expression in BMSCs decreased, which was reversed by the overexpression of IGF-1.

SFRP2 has a role in the regulation of Wnt/β-catenin signaling, and total cellular β-catenin and β-catenin localization was determined by Western blotting and IF staining, respectively. The Western blot results suggested that hypoxia decreased the total cellular β-catenin in both BMSCs-IGF-1 and BMSCs-NC. However, the level of total cellular β-catenin in BMSCs-IGF-1 was higher than in BMSCs-NC under both hypoxia and normoxia (Fig. 3a). Immunofluorescence staining results (Fig. 3b) were consistent with Western blot results. Higher nuclear levels of β-catenin were observed in BMSCs-IGF-1 compared with BMSCs-NC under both hypoxia and normoxia. Moreover, the expression levels of β-catenin targets cyclin D1 and c-Myc were also detected in both normoxic and hypoxic BMSCs. The data showed that cyclin D1 and c-Myc levels were higher in BMSCs-IGF-1 than in BMSCs-NC (Fig. 3a). These data suggested that overexpression of IGF-1 in BMSCs may activate AKT, SFRP2, and total cellular and nuclear β-catenin, resulting in the upregulation of cyclin D1 and c-Myc in either normoxic or hypoxic conditions.

### Overexpression of IGF-1 protects BMSCs against hypoxia via PI3K/AKT/SFRP2/β-catenin

**Fig. 4:**
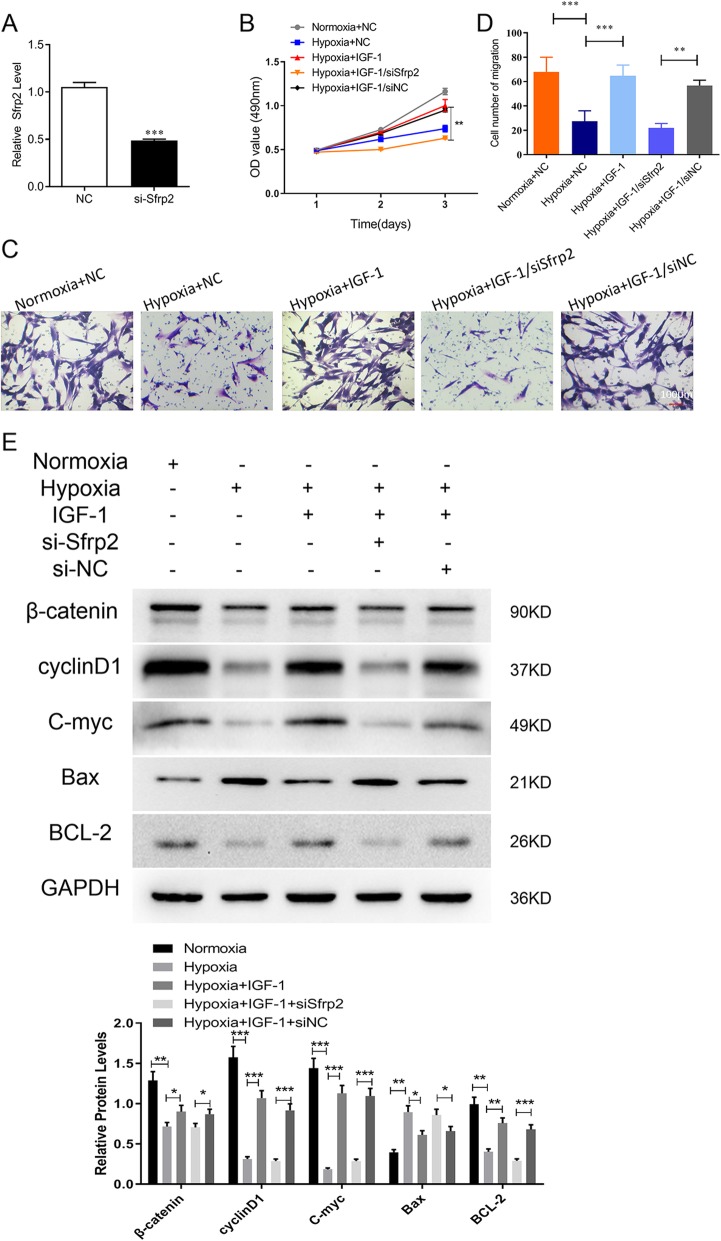
SFRP2 mediated BMSCs-IGF-1 resistance to hypoxia. BMSCs-IGF-1 were transfected with 100 nM si-*SFRP2* or si-NC, 48 h post-transfection; the cells were exposed to hypoxia for 48 h. **a** Expression of SFRP2 was measured by qPCR. **b** Cell proliferation was determined by MTS assay. **c**, **d** Cell migration was determined by Transwell assays. **e** Expression of β-catenin, cyclin D1, c-myc, BAX, and BCL-2 were determined by Western blotting. All assays were performed in triplicate (*P < 0.05, **P < 0.01, ***P < 0.001)

To explore whether SFRP2 is a major mediator of the resistance of BMSCs-IGF-1 to hypoxia, siRNA against *SFRP2* (si-*SFRP2*) was transfected into BMSCs-IGF-1 to knock down *SFRP2* prior to hypoxia exposure. Figure 4a showed that si-*SFRP2* could reduce the expression of *SFRP2*. As shown in Fig. 4b–d, compared with hypoxic BMSCs-NC, hypoxic BMSCs-IGF-1 showed a higher cell proliferation rate and migration abilities, while the resistance to hypoxia in BMSCs-IGF-1 was abolished by *SFRP2* knockdown. Moreover, the β-catenin accumulation and cyclin D1 and c-Myc expression in hypoxic BMSCs-IGF-1 were decreased by *SFRP2* knockdown (Fig. 4e). Additionally, apoptosis-related proteins were detected by Western blotting. A higher level of anti-apoptotic protein BCL-2 and a lower level of pro-apoptotic protein BAX were observed in hypoxic BMSCs-IGF-1 compared with BMSCs-NC. *SFRP2* knockdown abrogated these effects from the overexpression of IGF-1 under hypoxia (Fig. 4e).

**Fig. 5:**
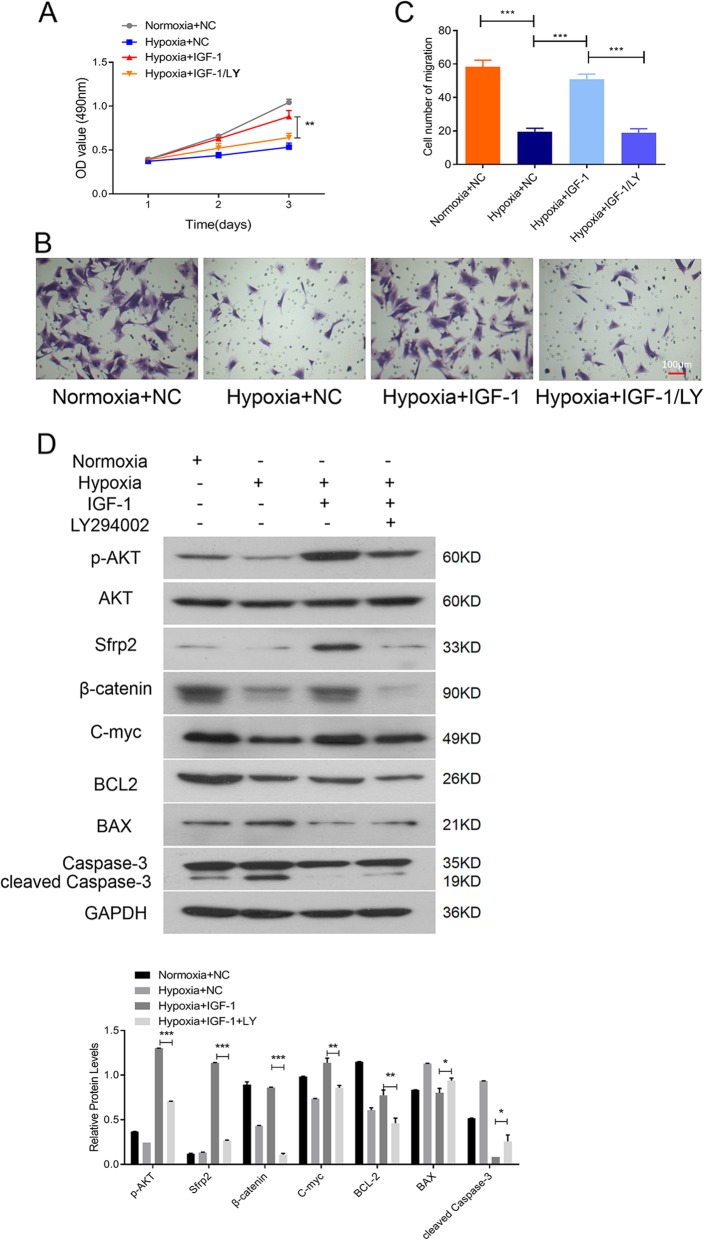
BMSCs-IGF-1 resistance to hypoxia is dependent on AKT. BMSCs-NC or BMSCs-IGF-1 were treated with 0.5 μM LY294002 or an equal volume of DMSO for 24 h, and then, the cells were exposed to hypoxia for 48 h. **a** Cell proliferation was determined by MTS assay. **b**, **c** Cell migration was determined by Transwell assay. **d** Expression of p-AKT, AKT, SFRP2, β-catenin, cyclin D1, c-myc, BAX, BCL-2, and cleaved caspase-3 were determined by Western blotting. All assays were performed in triplicate (*P < 0.05, **P < 0.01, ***P < 0.001)

LY294002, a strong inhibitor of phosphoinositide 3-kinases (PI3K), was employed to investigate whether IGF-1 overexpression protected hypoxic BMSCs via the activation of AKT. As shown in Fig. 5a–c, LY294002 pretreatment largely abrogated the protective effects of IGF-1 overexpression on cell proliferation and migration in hypoxic BMSCs. Moreover, LY294002 significantly counteracted the effects of IGF-1 on p-AKT, SFRP2, β-catenin, c-Myc, cleaved caspase-3, and BAX, and slightly downregulated BCL-2 in hypoxic BMSCs-IGF-1 (Fig. 5d).

### BMSCs-IGF-1 transplantation reduces infarct volume

To detect the main cardiac changes after MI, cardiac function and fibrosis were detected at 4 weeks later. Compared with the sham group, the area of fibrosis was significantly increased (Additional file 2: Figure S2A). The LVIDd and LVAWd were significantly increased, and EF, FS, SV, and CO were decreased in the MI group (Additional file 2: Figure S2B).

**Fig. 6:**
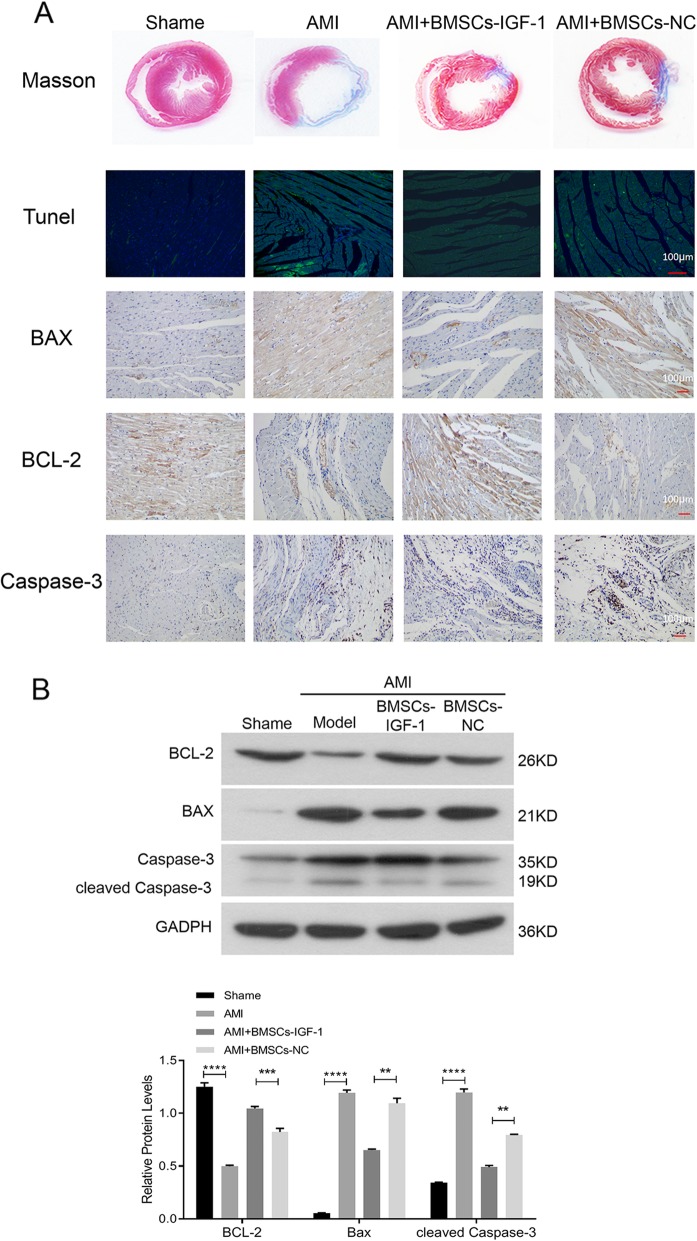
Effect of transplantation of BMSCs-IGF-1 following MI. **a** One week after coronary ligation, BMSCs-NC or BMSCs-IGF-1 (1 × 10^6^) were injected via the tail vein. Three weeks later, the fibrosis region (blue) was determined by Masson’s trichrome stain and the protein levels of BAX, BCL-2, and caspase-3 were determined by immunohistochemistry. **b** Expression of BAX, BCL-2, and cleaved caspase-3 were determined by Western blotting (*P < 0.05, **P < 0.01, ***P < 0.001)

We assessed the cytoprotective effects of BMSCs-IGF-1 in vivo by injecting 1 × 10^6^ BMSCs-IGF-1 or BMSCs-NC through the tail vein 1 week after coronary ligation. At 3 weeks after injections, rats were sacrificed, and the hearts were harvested for histological and Western blot analysis. Immunofluorescence staining showed that the number of BMSCs-IGF-1 cells is more retented and survived in vivo than that in BMSCs-NC group (Additional file 3: Figure S3). Masson’s trichrome staining indicated that the area of fibrosis was reduced by BMSCs-NC transplantation compared with blank control. Furthermore, BMSCs-IGF-1 injection significantly reduced the area of fibrosis compared with BMSCs-NC (Fig. 6a). The TUNEL and IHC assay suggested that apoptosis in the hearts of rats injected with BMSCs-IGF-1 was lower than in the BMSCs-NC and control groups (Fig. 6a). Moreover, the apoptosis-related proteins in the rat hearts in each group were determined by Western blotting. As shown in Fig. 6b, in BMSCs-IGF-1-transplanted rat hearts, decreased BAX and cleaved caspase-3, and increased BCL-2 levels were observed compared with the BMSCs-NC and control groups. These results implied that BMSCs-IGF-1 transplantation could upregulate anti-apoptotic and downregulate pro-apoptotic signals in MI tissues to promote cardiac repair and reduce fibrosis area.

## Discussion

BMSC has been considered as a promising candidate cell type in stem cell-based therapy for AMI due to its ease of preparation, potential differentiation into a cardiac lineage, and paracrine secretion of growth factors [23]. However, the clinical effects of BMSC therapy are limited by the poor survival of transplanted stem cells in the hostile microenvironment of the ischemic region [24, 25]. This warrants the need to develop genetically modified BMSCs that can maintain high cell viability under hypoxic conditions and improve the microenvironment of transplanted zones.

In the present study, genetically engineered BMSCs overexpressing IGF-1 were used to enhance resistance to hypoxia-induced apoptosis and improve the therapeutic efficacy for AMI. The major novel findings in the present work are as follows: (1) IGF-1 could improve BMSC proliferation and resistance to hypoxia, (2) the effects of IGF-1 on the properties of BMSCs are at least partly mediated by the AKT/SFRP2 pathway, (3) in vitro and in vivo studies revealed that IGF-1 overexpression in BMSCs can further reduce apoptosis of cardiomyocytes under hypoxia, and most importantly, (4) we found that SFRP2 is an important mediator for enhancing BMSC viability by acting as an agonist rather than as an antagonist in the Wnt/β-catenin pathway.

It has been reported that AKT-modified BMSCs mediated pro-survival effects in vitro as well as the diminution of infarction areas and the restoration of cardiac function in MI rodent hearts via the secretion of SFRP2 [8]. Based on these findings, we attempted to construct genetically engineered BMSCs that could maintain high cell viability under hypoxia and release pro-survival factors to modify the hostile environment. In our study, BMSCs-IGF-1 could secret fourfold more IGF-1 and exhibited a higher cell proliferation rate and migrating ability, more stemness, and resistance to apoptosis under hypoxia compared with BMSCs-NC. Inhibition of AKT or SFRP2 significantly antagonized these effects. These findings supported our hypothesis that the effects of IGF-1 on BMSCs are mediated by the AKT/SFRP2 signaling pathway.

To evaluate the protective effects of BMSCs-IGF-1 on cardiomyoblasts, H9C2 was co-cultured with BMSCs-IGF-1 or BMSCs-NC. The results showed that BMSCs-IGF-1 could rescue cardiomyoblasts from apoptosis and enhance cell viability of H9C2 under hypoxia. Cselenyák et al. reported that BMSCs could rescue cardiomyoblasts from cell death in an in vitro ischemia model, in a process that was dependent on direct cell-to-cell connections, and the physical separation of the two cell populations abolished the protective effects of BMSCs [26]. In our experiments, co-culture was performed with cell culture inserts that physically separated two cell populations growing in the same medium. Impressively, BMSCs-IGF1-1 showed protective effects on H9C2 under hypoxia through a mechanism that was independent of cell-to-cell connections, indicating that the mediator was likely to be soluble paracrine factors. These results further supported the notion that the effects of BMSCs on treatment for AMI may be largely mediated by their paracrine effects [27]. Our in vivo results further confirmed the protective effects of BMSCs-IGF-1 in AMI hearts. Transplantation of BMSCs-IGF-1 significantly increased BCL-2 and downregulated BAX and caspase-3 expression, thereby depressing apoptosis in cardiomyocytes, and resulting in a further decrease in infarction area size compared with the BMSCs-NC transplantation.

Wnt/β-catenin signaling is highly conserved and plays an important role in stem cell proliferation and differentiation. When the Wnt ligand binds to the frizzled/low-density lipoprotein receptor-related protein (LRP) receptor complex, the Wnt/β-catenin cascade is activated, leading to the accumulation of β-catenin. Conversely, when the Wnt ligands are absent, or the frizzled/LRP receptor complex is blocked by the antagonists, cytoplasmic β-catenin is subsequently recruited to the destruction complex (DC) for degradation [28]. SFRP2 has been viewed as an antagonist of the Wnt/β-catenin pathway due to its homology with the extracellular portion of the Wnt receptor frizzled. However, in our study, we found that in BMSCs-IGF-1, both cellular and nuclear levels of β-catenin were increased, coupled with an increased expression of SFRP2. Inhibition of AKT or *SFRP2* knockdown by siRNA significantly antagonized the effect of IGF-1 and decreased the expression of β-catenin. Furthermore, the protein expression of β-catenin target genes (cyclin D1 and c-Myc) were accordingly changed. These novel findings suggest that in IGF-1-overexpressing BMSCs, the secreted factor SFRP2 is an important mediator for enhancing stem cell viability via activating, rather than antagonizing, the Wnt/β-catenin pathway. Other studies also demonstrated that SFPR2 may be coupled with Wnt-activating activity in different cell types or organ systems [16, 17, 29–31]. Lin et al. showed that in cardiac fibroblasts, SFRP2 activated cell proliferation and energy metabolism in part through Wnt/β-catenin signaling [17]. Esteve et al. demonstrated that in vertebrate optic cup development, SFRP2 was required for Wnt/β-catenin signaling activation [29]. Furthermore, Hasebe et al. reported that the thyroid hormone could upregulate the expression of SFRP2, activating Wnt/β-catenin signaling and c-Myc expression in the post-embryonic intestine to promote adult stem cell proliferation [16]. The underlying mechanisms for SFRP2 in activating Wnt/β-catenin signaling have not been fully elucidated. It has been proposed that SFRP2 can modulate frizzled receptor-mediated signaling independently of Wnt ligands, or form complexes with both frizzled receptor and Wnt ligands through differential domain binding [29, 32].

There are several limitations in our study. Due to limited funding, we did not detect the role of exosome during the interaction between BMSCs-IGF-1 and H9C2 cardiomyoblast cells, which would be interested for exploring the paracrine effects of BMSCs-IGF-1 on treatment for AMI. Second, in this study, we designed the in vivo experiments to detect the improvement of cardiac function after BMSC treatment. The molecules of signaling pathway such as Sfrp2, AKT, and β-catenin were not detected.

## Conclusions

In conclusion, our study suggests BMSCs-IGF-1 exhibit a higher cell proliferation rate, migrating ability, more stemness and resistance to apoptosis, and more protective effects on cardiomyoblasts under hypoxia compared with BMSCs-NC. These effects were mediated by the AKT/SFRP2 axis. SFRP2 serves as an agonist rather than as an antagonist of the Wnt/β-catenin pathway in this cellular context. Transplantation of BMSCs-IGF-1 results in better cardiac repair and regeneration after MI. These findings indicate that the IGF-1/SFRP2/β-catenin axis might be an effective therapeutic target for stem cell therapy of cardiovascular diseases.

## Supplementary information

**Additional file 1:** **Figure S1.** The separated and merged images of TUNEL assay for accessment of IGF-1 overexpression on apoptosis of BMSCs. Apoptotic cells were labeled with fluorescein-12-dUTP, resulting in localized green fluorescence within the nuclei. The number of TUNEL-positive cells was counted in a blind fashion.

**Additional file 2:** **Figure S2.** Establishment of rat myocardial infarction model and verification. (A) Masson’s trichrome staining of heart sections and representative echocardiograms at 4 weeks after MI are shown. (B). The ejection fraction (EF), fractional shortening (FS), left ventricle inner diameter during diastole (LVID d), left ventricle anterior wall thickness during diastole (LVAW d), cardiac output (CO), and stroke volume (SV) were measured. *n* = 6 per group (*P < 0.05, **P < 0.01, *** *P* < 0.001).

**Additional file 3:** **Figure S3.** Distribution of BMSC-IGF-1 and BMSC-NC after transplantation into the rats with myocardial infarction. The GFP-positive cells (green fluorescence) under immunofluorescence staining indicated BMSCs (arrow), and blue fluorescence represented DAPI staining for cardiomyocytes. The quantitative analysis were measured by ImageJ software. Original magnifications 200X (**P < 0.01).

## Data Availability

The datasets generated and/or analyzed during the current study are available from the corresponding author on reasonable request.

## Abbreviations

AMI: Acute myocardial infarction
BMSC: Bone marrow mesenchymal stem cell
CD29: Cluster of differentiation 29
CD45: Cluster of differentiation 45
CD73: Cluster of differentiation 73
CD90: Cluster of differentiation 90
CD105: Cluster of differentiation 105
CHF: Chronic heart failure
CO: Cardiac output
DC: Destruction complex
DMEM: Dulbecco’s modified Eagle’s medium
EF: Ejection fraction
ELISA: Enzyme-linked immunosorbent assay
FBS: Fetal bovine serum
FS: Fractional shortening
IF: Immunofluorescence
IGF-1: Insulin-like growth factor-1
IHC: Immunohistochemistry
LRP: Lipoprotein receptor related protein
LVAWd: Left ventricular anterior wall during diastole
LVIDd: Left ventricular internal diameter at end diastole
MI: Myocardial infarction
MTS: 3-(4,5-Dimethylthiazol-2-yl)-5-(3-carboxymethoxyphenyl)-2-(4-sulfophenyl)-2h-tetrazolium
PFA: Paraformaldehyde
PI3K: Phosphoinositide 3-kinases
SD: Sprague-Dawley
SFRP2: Secreted frizzled-related protein 2
SV: Stroke volume
TUNEL: Terminal deoxynucleotidyl transferase-mediated dUTP nick-end labeling

## References

[1] Wu J, Hall M, Dondo TB, Wilkinson C, Ludman P, DeBelder M (2019). Association between time of hospitalization with acute myocardial infarction and in-hospital mortality. Eur Heart J.

[2] Blau HM, Daley GQ (2019). Stem cells in the treatment of disease. N Engl J Med.

[3] Goradel NH, Hour FG, Negahdari B, Malekshahi ZV, Hashemzehi M, Masoudifar A (2018). Stem cell therapy: a new therapeutic option for cardiovascular diseases. J Cell Biochem.

[4] Harrell Carl Randall, Fellabaum Crissy, Jovicic Nemanja, Djonov Valentin, Arsenijevic Nebojsa, Volarevic Vladislav (2019). Molecular Mechanisms Responsible for Therapeutic Potential of Mesenchymal Stem Cell-Derived Secretome. Cells.

[5] Fan M, Huang Y, Chen Z, Xia Y, Chen A, Lu D (2019). Efficacy of mesenchymal stem cell therapy in systolic heart failure: a systematic review and meta-analysis. Stem Cell Res Ther.

[6] Hu X, Yu SP, Fraser JL, Lu Z, Ogle ME, Wang JA (2008). Transplantation of hypoxia-preconditioned mesenchymal stem cells improves infarcted heart function via enhanced survival of implanted cells and angiogenesis. J Thorac Cardiovasc Surg.

[7] Gnecchi M, He H, Melo LG, Noiseaux N, Morello F, de Boer RA (2009). Early beneficial effects of bone marrow-derived mesenchymal stem cells overexpressing Akt on cardiac metabolism after myocardial infarction. Stem Cells.

[8] He W, Zhang L, Ni A, Zhang Z, Mirotsou M, Mao L (2010). Exogenously administered secreted frizzled related protein 2 (Sfrp2) reduces fibrosis and improves cardiac function in a rat model of myocardial infarction. Proc Natl Acad Sci U S A.

[9] Golpanian S, Wolf A, Hatzistergos KE, Hare JM (2016). Rebuilding the damaged heart: mesenchymal stem cells, cell-based therapy, and engineered heart tissue. Physiol Rev.

[10] Allahdadi KJ, de Santana TA, Santos GC, Azevedo CM, Mota RA, Nonaka CK (2019). IGF-1 overexpression improves mesenchymal stem cell survival and promotes neurological recovery after spinal cord injury. Stem Cell Res Ther.

[11] Huang YL, Kuang J, Hu YZ, Song YB, Qiu RF, Mai WY (2012). Bone marrow stromal cell transplantation combined with angiotensin-converting enzyme inhibitor treatment in rat with acute myocardial infarction and the role of insulin-like growth factor-1. Cytotherapy..

[12] Huang YL, Qiu RF, Mai WY, Kuang J, Cai XY, Dong YG (2012). Effects of insulin-like growth factor-1 on the properties of mesenchymal stem cells in vitro. J Zhejiang Univ Sci B.

[13] Janda CY, Dang LT, You C, Chang J, de Lau W, Zhong ZA (2017). Surrogate Wnt agonists that phenocopy canonical Wnt and beta-catenin signalling. Nature..

[14] Hang K, Ye C, Xu J, Chen E, Wang C, Zhang W (2019). Apelin enhances the osteogenic differentiation of human bone marrow mesenchymal stem cells partly through Wnt/beta-catenin signaling pathway. Stem Cell Res Ther.

[15] Liu Y, Zhou Q, Zhou D, Huang C, Meng X, Li J (2017). Secreted frizzled-related protein 2-mediated cancer events: friend or foe?. Pharmacol Rep.

[16] Hasebe T, Fujimoto K, Kajita M, Ishizuya-Oka A (2016). Thyroid hormone activates Wnt/beta-catenin signaling involved in adult epithelial development during intestinal remodeling in Xenopus laevis. Cell Tissue Res.

[17] Lin H, Angeli M, Chung KJ, Ejimadu C, Rosa AR, Lee T (2016). sFRP2 activates Wnt/beta-catenin signaling in cardiac fibroblasts: differential roles in cell growth, energy metabolism, and extracellular matrix remodeling. Am J Physiol Cell Physiol.

[18] Dominici M, Le Blanc K, Mueller I, Slaper-Cortenbach I, Marini F, Krause D (2006). Minimal criteria for defining multipotent mesenchymal stromal cells. The International Society for Cellular Therapy position statement. Cytotherapy..

[19] Zhang P, Xing C, Rhodes SD, He Y, Deng K, Li Z (2016). Loss of Asxl1 alters self-renewal and cell fate of bone marrow stromal cell, leading to Bohring-Opitz-like syndrome in mice. Stem Cell Rep.

[20] Piccinato CA, Sertie AL, Torres N, Ferretti M, Antonioli E (2015). High OCT4 and low p16(INK4A) expressions determine in vitro lifespan of mesenchymal stem cells. Stem Cells Int.

[21] Mirotsou M, Zhang Z, Deb A, Zhang L, Gnecchi M, Noiseux N (2007). Secreted frizzled related protein 2 (Sfrp2) is the key Akt-mesenchymal stem cell-released paracrine factor mediating myocardial survival and repair. Proc Natl Acad Sci U S A.

[22] Zhang Z, Deb A, Zhang Z, Pachori A, He W, Guo J (2009). Secreted frizzled related protein 2 protects cells from apoptosis by blocking the effect of canonical Wnt3a. J Mol Cell Cardiol.

[23] Yun Chul, Lee Sang (2019). Enhancement of Functionality and Therapeutic Efficacy of Cell-Based Therapy Using Mesenchymal Stem Cells for Cardiovascular Disease. International Journal of Molecular Sciences.

[24] Shafei AE, Ali MA, Ghanem HG, Shehata AI, Abdelgawad AA, Handal HR (2018). Mechanistic effects of mesenchymal and hematopoietic stem cells: new therapeutic targets in myocardial infarction. J Cell Biochem.

[25] Roura S, Galvez-Monton C, Mirabel C, Vives J, Bayes-Genis A (2017). Mesenchymal stem cells for cardiac repair: are the actors ready for the clinical scenario?. Stem Cell Res Ther.

[26] Cselenyak A, Pankotai E, Horvath EM, Kiss L, Lacza Z (2010). Mesenchymal stem cells rescue cardiomyoblasts from cell death in an in vitro ischemia model via direct cell-to-cell connections. BMC Cell Biol.

[27] Abushouk AI, Salem A, Saad A, Afifi AM, Afify AY, Afify H (2019). Mesenchymal stem cell therapy for doxorubicin-induced cardiomyopathy: potential mechanisms, governing factors, and implications of the heart stem cell debate. Front Pharmacol.

[28] Zeng X, Zhang Y, Xu H, Zhang T, Xue Y, An R (2018). Secreted frizzled related protein 2 modulates epithelial-mesenchymal transition and stemness via Wnt/beta-catenin signaling in choriocarcinoma. Cell Physiol Biochem.

[29] Esteve P, Sandonis A, Ibanez C, Shimono A, Guerrero I, Bovolenta P (2011). Secreted frizzled-related proteins are required for Wnt/beta-catenin signalling activation in the vertebrate optic cup. Development..

[30] Kwack MH, Ahn JS, Jang JH, Kim JC, Sung YK, Kim MK (2016). SFRP2 augments Wnt/beta-catenin signalling in cultured dermal papilla cells. Exp Dermatol.

[31] von Marschall Z, Fisher LW (2010). Secreted frizzled-related protein-2 (sFRP2) augments canonical Wnt3a-induced signaling. Biochem Biophys Res Commun.

[32] Bovolenta P, Esteve P, Ruiz JM, Cisneros E, Lopez-Rios J (2008). Beyond Wnt inhibition: new functions of secreted frizzled-related proteins in development and disease. J Cell Sci.

