# Supplementary figures and images for: IGF-1 enhances BMSC viability, migration, and anti-apoptosis in myocardial infarction via secreted frizzled-related protein 2 pathway
Source: Stem Cell Res Ther. 2020 Jan 9;11:22. doi: 10.1186/s13287-019-1544-y (PMC6953226; doi:10.1186/s13287-019-1544-y)

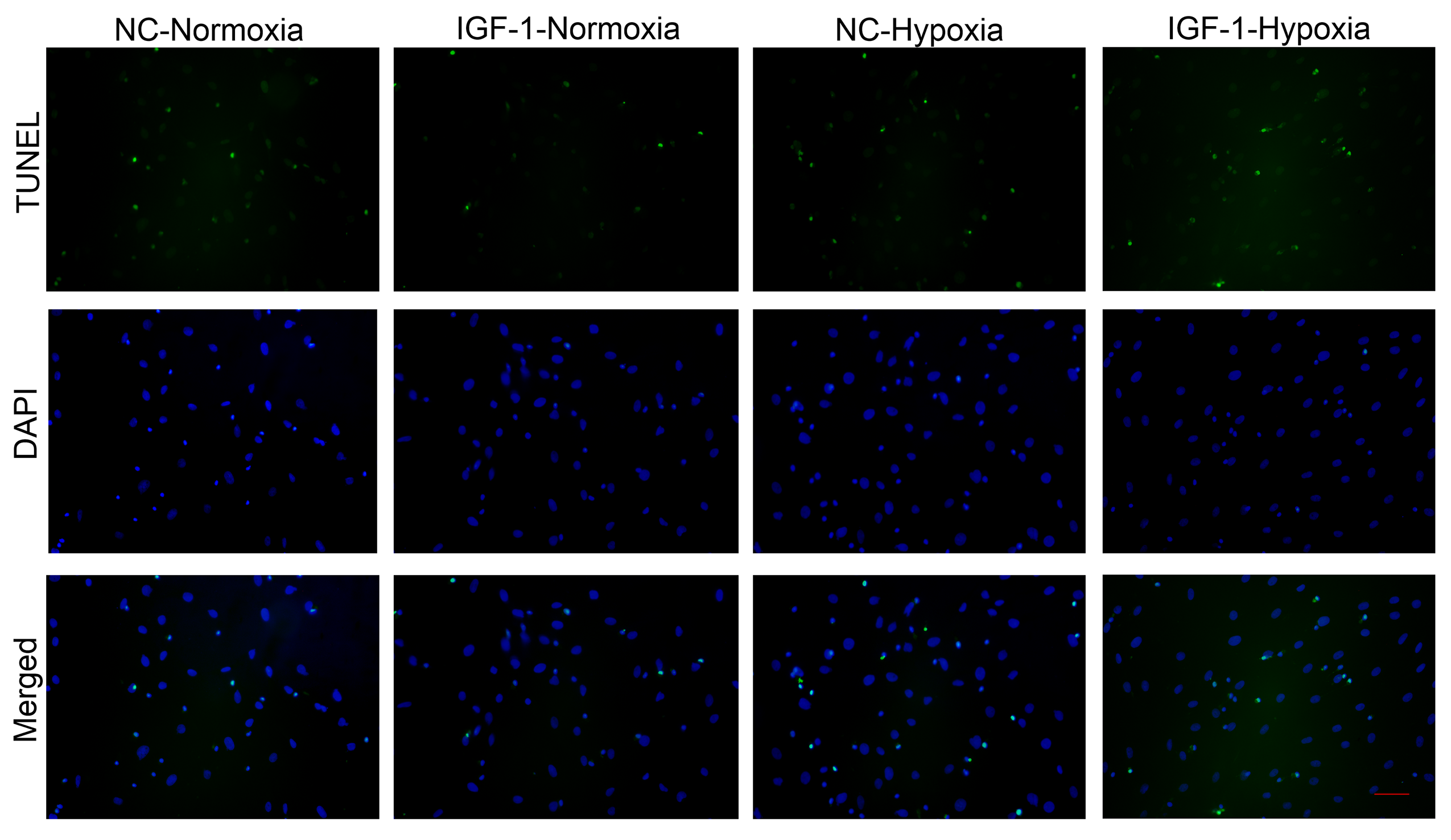

Supplement: Supplementary file 1 — Additional file 1: Figure S1. The separated and merged images of TUNEL assay for accessment of IGF-1 overexpression on apoptosis of BMSCs. Apoptotic cells were labeled with fluorescein-12-dUTP, resulting in localized green fluorescence within the nuclei. The number of TUNEL-positive cells was counted in a blind fashion. [file 13287_2019_1544_MOESM1_ESM.tif]

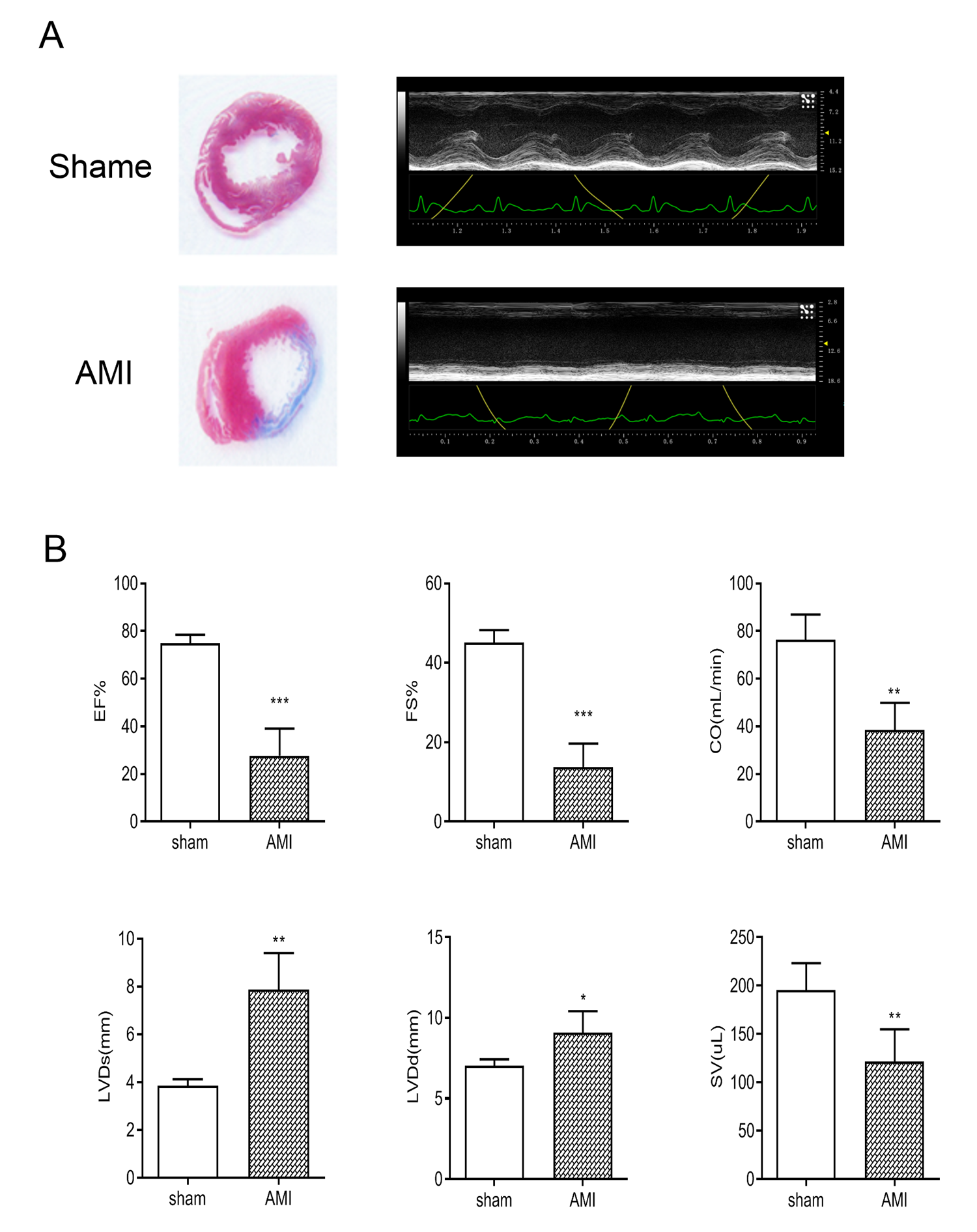

Supplement: Supplementary file 2 — Additional file 2: Figure S2. Establishment of rat myocardial infarction model and verification. (A) Masson’s trichrome staining of heart sections and representative echocardiograms at 4 weeks after MI are shown. (B). The ejection fraction (EF), fractional shortening (FS), left ventricle inner diameter during diastole (LVID d), left ventricle anterior wall thickness during diastole (LVAW d), cardiac output (CO), and stroke volume (SV) were measured. n = 6 per group (*P < 0.05, **P < 0.01, *** P < 0.001). [file 13287_2019_1544_MOESM2_ESM.tif]

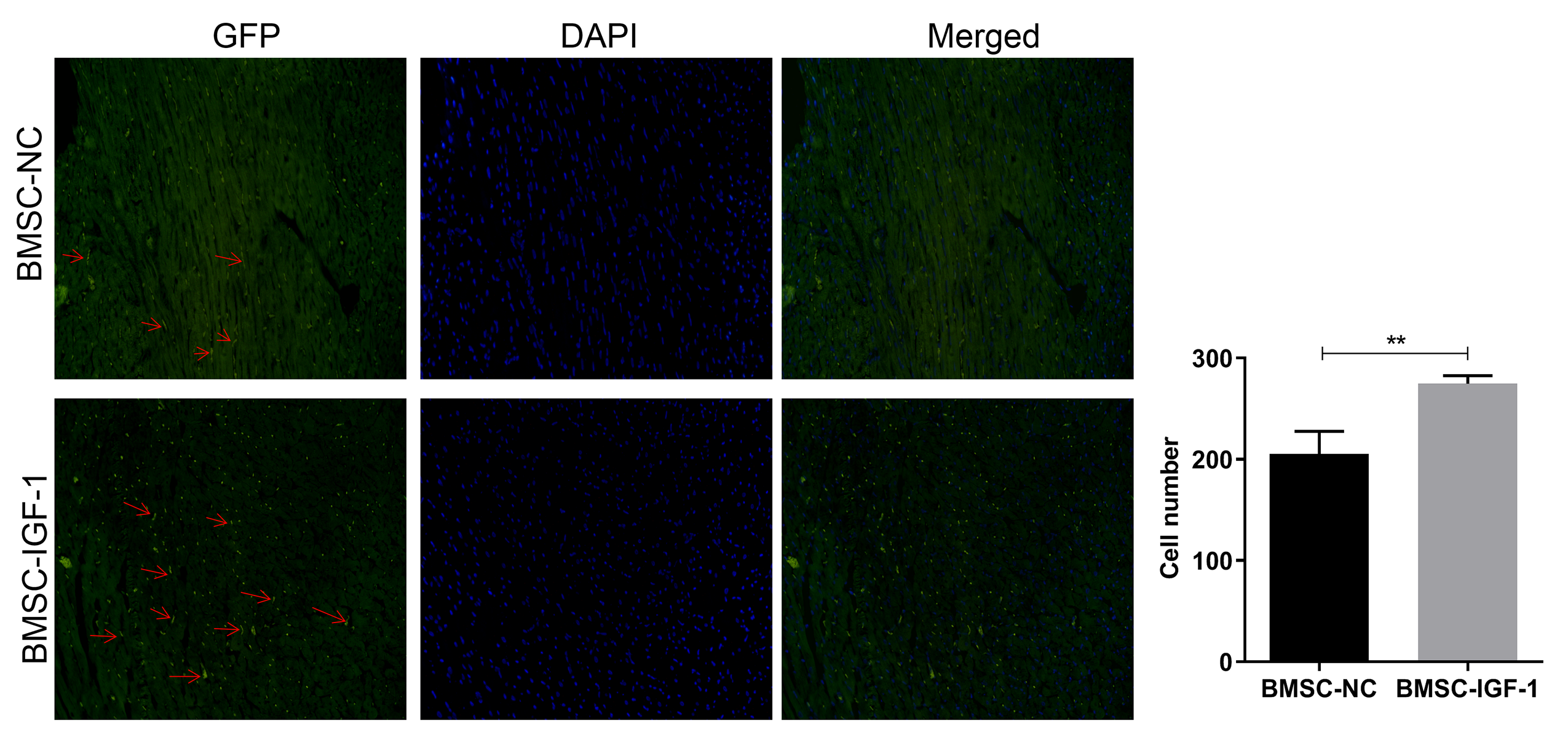

Supplement: Supplementary file 3 — Additional file 3: Figure S3. Distribution of BMSC-IGF-1 and BMSC-NC after transplantation into the rats with myocardial infarction. The GFP-positive cells (green fluorescence) under immunofluorescence staining indicated BMSCs (arrow), and blue fluorescence represented DAPI staining for cardiomyocytes. The quantitative analysis were measured by ImageJ software. Original magnifications 200X (**P < 0.01). [file 13287_2019_1544_MOESM3_ESM.tif]
